# P44 Multi-source environmental reservoirs drive *Enterobacter cloacae* complex transmission: genomic evidence from an LMIC hospital outbreak investigation

**DOI:** 10.1093/jacamr/dlaf230.051

**Published:** 2025-12-04

**Authors:** M Nizam Ahmed, Mamta Puraswani, Parul Singh, Bharat Chandra Das, Vanlal Tluanpuii, Madhavi Kirti, Subodh Kumar, Sushma Sagar, Kapil Dev Soni, Richa Aggarwal, Ashish Bindra, Keshav Goyal, Gyanendra Pal Singh, Navdeep Sokhal, Kamran Farooque, Purva Mathur

**Affiliations:** All India Institute of Medical Sciences, New Delhi, India; Trauma Centre, All India Institute of Medical Sciences, New Delhi, India; All India Institute of Medical Sciences, New Delhi, India; All India Institute of Medical Sciences, New Delhi, India; All India Institute of Medical Sciences, New Delhi, India; All India Institute of Medical Sciences, New Delhi, India; All India Institute of Medical Sciences, New Delhi, India; All India Institute of Medical Sciences, New Delhi, India; Trauma Centre, All India Institute of Medical Sciences, New Delhi, India; Trauma Centre, All India Institute of Medical Sciences, New Delhi, India; Trauma Centre, All India Institute of Medical Sciences, New Delhi, India; Trauma Centre, All India Institute of Medical Sciences, New Delhi, India; Trauma Centre, All India Institute of Medical Sciences, New Delhi, India; Trauma Centre, All India Institute of Medical Sciences, New Delhi, India; Trauma Centre, All India Institute of Medical Sciences, New Delhi, India; All India Institute of Medical Sciences, New Delhi, India

## Abstract

**Background:**

The *Enterobacter cloacae* complex (ECC) is increasingly recognized as a major opportunistic pathogen in healthcare-associated infections (HAIs), particularly in intensive care settings. Its clinical relevance has risen in parallel with the global spread of MDR strains, including carbapenemase producers, which complicate management and contribute to excess morbidity, prolonged hospitalization and increased healthcare costs. Outbreaks caused by ECC are often traced to contaminated water systems, medical devices, or the hands of healthcare workers, but reports from low and middle income countries (LMICs) remain scarce. In August–September 2023, a cluster of ECC infections was detected at a Level-1 trauma centre in northern India. An urgent epidemiological and microbiological investigation was undertaken to delineate transmission dynamics, identify reservoirs and guide infection prevention and control (IPC) measures.

**Methods:**

We conducted a prospective outbreak investigation of all culture-confirmed ECC cases occurring ≥48 h after admission during August–September 2023. Cases were identified in real time and reported in parallel with implementation of control measures. Clinical and epidemiological data included demographics, comorbidities, prior antibiotic exposure, invasive device use and outcomes. Environmental surveillance targeted high-risk areas such as water sources, sinks, taps and hand swabs from healthcare workers. Antimicrobial susceptibility testing (AST) was performed using standard protocols. Infection prevention and control (IPC) interventions included reinforcement of hand hygiene, environmental decontamination, chlorination and treatment of hospital water systems, and central line-associated bloodstream infection (CLABSI) care bundles. WGS was undertaken on 10 representative isolates (5 clinical, 5 environmental) to determine clonal relatedness, sequence types, plasmid profiles and antimicrobial resistance

**Results:**

A total of 19 developed ECC infections; 63% were admitted to the ICU. No deaths occurred. Prior antibiotic exposure was reported in 68% and invasive procedures in 74%. Median ICU stay among infected patients was 14 days compared to 8 days in non-ICU cases (*P*=0.03). Environmental surveillance yielded 52 ECC isolates: 60% from healthcare worker hand swabs and 40% from hospital water samples. All isolates were resistant to cefuroxime. 80% were susceptible to amikacin, meropenem, imipenem, cefepime, tigecycline and cotrimoxazole. WGS showed clonal dissemination of ST97 (60%) and ST171 (40%) with >99.8% identity between clinical and environmental isolates. Plasmids IncX3_1 and IncFII(pECLA)_1_pECLA carried *bla*_NDM_, *bla*_ACT-5_ and *bla*_SHV_. Genes *oqxA*/*oqxB* (100%) and *fosA* (68%) were detected. Following IPC interventions, ECC incidence decreased from 15.8 to 6.7 cases per 1000 patient-days (*P<*0.05). Water contamination fell from 40% to 10%, and hand hygiene compliance improved from 60% to 85%. No additional cases occurred after September 2023.

**Conclusions:**

This outbreak was driven by clonal dissemination of ECC, primarily through water systems and healthcare worker hands. WGS provided high resolution evidence linking clinical and environmental isolates, enabling targeted interventions. The multimodal IPC response including reinforced hand hygiene, water decontamination and central line care bundles successfully reduced transmission and contained the outbreak. However, residual water contamination highlights the resilience of ECC in biofilm-associated reservoirs. Our findings underscore the value of integrating genomic epidemiology into outbreak investigations in LMIC settings and the need for sustained environmental monitoring.

